# Patterning Biomaterials for the Spatiotemporal Delivery of Bioactive Molecules

**DOI:** 10.3389/fbioe.2016.00045

**Published:** 2016-06-02

**Authors:** Silvia Minardi, Francesca Taraballi, Laura Pandolfi, Ennio Tasciotti

**Affiliations:** ^1^Department of Regenerative Medicine, Houston Methodist Research Institute, Houston, TX, USA; ^2^College of Materials Science and Engineering, University of Chinese Academy of Science, Beijing, China; ^3^Department of Orthopedics, Houston Methodist Hospital, Houston, TX, USA

**Keywords:** biomaterials, patterning, tissue engineering, growth factors, drug delivery

## Abstract

The aim of tissue engineering is to promote the repair of functional tissues. For decades, the combined use of biomaterials, growth factors (GFs), and stem cells has been the base of several regeneration strategies. Among these, biomimicry emerged as a robust strategy to efficiently address this clinical challenge. Biomimetic materials, able to recapitulate the composition and architecture of the extracellular matrix, are the materials of choice, for their biocompatibility and higher rate of efficacy. In addition, it has become increasingly clear that restoring the complex biochemical environment of the target tissue is crucial for its regeneration. Toward this aim, the combination of scaffolds and GFs is required. The advent of nanotechnology significantly impacted the field of tissue engineering by providing new ways to reproduce the complex spatial and temporal biochemical patterns of tissues. This review will present the most recent approaches to finely control the spatiotemporal release of bioactive molecules for various tissue engineering applications.

## Introduction

The use of autologous or heterologous cells in clinical practice has always been considered the most advantageous strategy for boosting tissue repair (Jaklenec et al., 2012). However, several downsides, such as their costs, availability, risk of infection, pain, and low viability after injection, subvert their advantages (Harrison et al., 2014). Thus, the ability to elicit specific cell responses *in vivo* through the release of bioactive signaling molecules has attracted increasing attention.

Growth factors (GFs) are soluble molecules that control a wide range of signaling pathways by binding to specific cell receptors (Vo et al., 2012). In 2010, therapeutics based on bioactive proteins and peptides represented about 13% of global sales in the biomedical field (Sheridan, 2010). Furthermore, sales have been predicted to increase from $14.1 billion in 2011 to $25.4 billion by 2018 (Fosgerau and Hoffmann, 2015).

The biological response triggered by a GF depends on the target cells, their number, and other signaling factors present in the *milieu* of a specific tissue. Therefore, the successful use of GFs in regenerative therapies requires the selection of appropriate GFs to accomplish optimal tissue repair. Toward this end, the precise regulation of GFs’ concentration in space and time is vital (Guldberg, 2009).

In order to increase functional regeneration, many proposed approaches combine surgical procedures, biologics, and biomaterials (Fisher and Mauck, 2013). Biomaterials (derived from natural or synthetic sources) are contributing to groundbreaking work in many tissue engineering applications (Balint et al., 2014), including bone and cartilage regeneration (Tampieri et al., 2008; Henkel et al., 2013; Minardi et al., 2015). The biomaterials market for implantable devices is estimated to be worth $33 billion by the end of 2019 (Transparency Market Research, 2012). Tissue healing in musculoskeletal surgery has benefited from the surgical implantation of bio-conductive scaffolds (Hsu et al., 2012). However, the use of GFs and chemo/cytokines in clinical practice yielded controversial results (Garner et al., 2011; Anitua et al., 2012). One famous example involved the use of recombinant human bone morphogenetic protein-2 (rhBMP-2) in spinal fusion (Medtronic, INFUSE^®^: rhBMP-2-infused collagen scaffold). Although approved by the US Food and Drug Administration (FDA) in 2002, the high doses of rhBMP-2 used by this device resulted in major adverse side effects, including cancer, spinal cord compression from soft tissue swelling, spinal cord impingement from ectopic bone formation, elevated bone resorption from osteoclast activation, and preferential induction of adipogenesis over osteogenesis (Kaneko et al., 2000; Smucker et al., 2006; Wong et al., 2008; Robin et al., 2010; Zara et al., 2011; Epstein, 2013).

These side effects were caused by the massive release of rhBMP-2 from the collagen sponge after implantation (5–10 mg of BMP-2/implant) (McKay et al., 2007). This dose is over 5000 times higher than the amount required for bone formation *in vitro* (Wang et al., 1988; Place et al., 2009) and even higher than physiologic doses found in the osteogenic niche where other osteogenic stimuli (chemical, physical, and structural) additionally contribute to induce bone formation (Place et al., 2009; Tampieri et al., 2011). When the uncontrolled pharmacokinetics showed adverse side effects, the FDA revoked the device’s approval (McKie et al., 2014).

After this, GF dose–response effects became all the more crucial (Shields et al., 2006), and it became evident that adverse outcomes could have been avoided with the controlled, localized release of rhBMP-2. It was determined that burst release and widespread tissue exposure to GFs, together with an early dissipation from the implantation site (e.g., with irrigation, bleeding, and edema) were not ideal pharmacokinetics for tissue regeneration (Lai et al., 2013). Furthermore, it has become clear that the first-order release of a single bioactive molecule is not sufficient to mimic the complex biochemical gradients present at a specific stage of tissue regeneration (Wang et al., 2013; Minardi et al., 2014).

Extraordinary progress has been made toward the design of biomaterials with suitable multiscale hierarchical structures, facilitating the staged release of a combination of bioactive molecules, according to any complex delivery pattern (Biondi et al., 2008; Guldberg, 2009; Chen et al., 2010).

Bioactive factors can be incorporated within materials using layer deposition or integrated into their fibrous mesh *via* electrospinning or self-assembly techniques (Sun et al., 2003; Hosseinkhani et al., 2006; Minardi et al., 2014). Herein, we review the most recent strategies for biomaterial fabrication, featuring spatiotemporal patterns of bioactive molecules.

## Engineering 3D Biopatterned Materials

The three-dimensional (3D) interactions between cells and the extracellular matrix (ECM) have been proven to be crucial to orchestrate tissue formation and regeneration in response to injury. Materials such as hydrogels and scaffolds engineered to emulate the ECM can support tissue healing (Hoffman, 2012; Loh and Choong, 2013).

**Figure 1:**
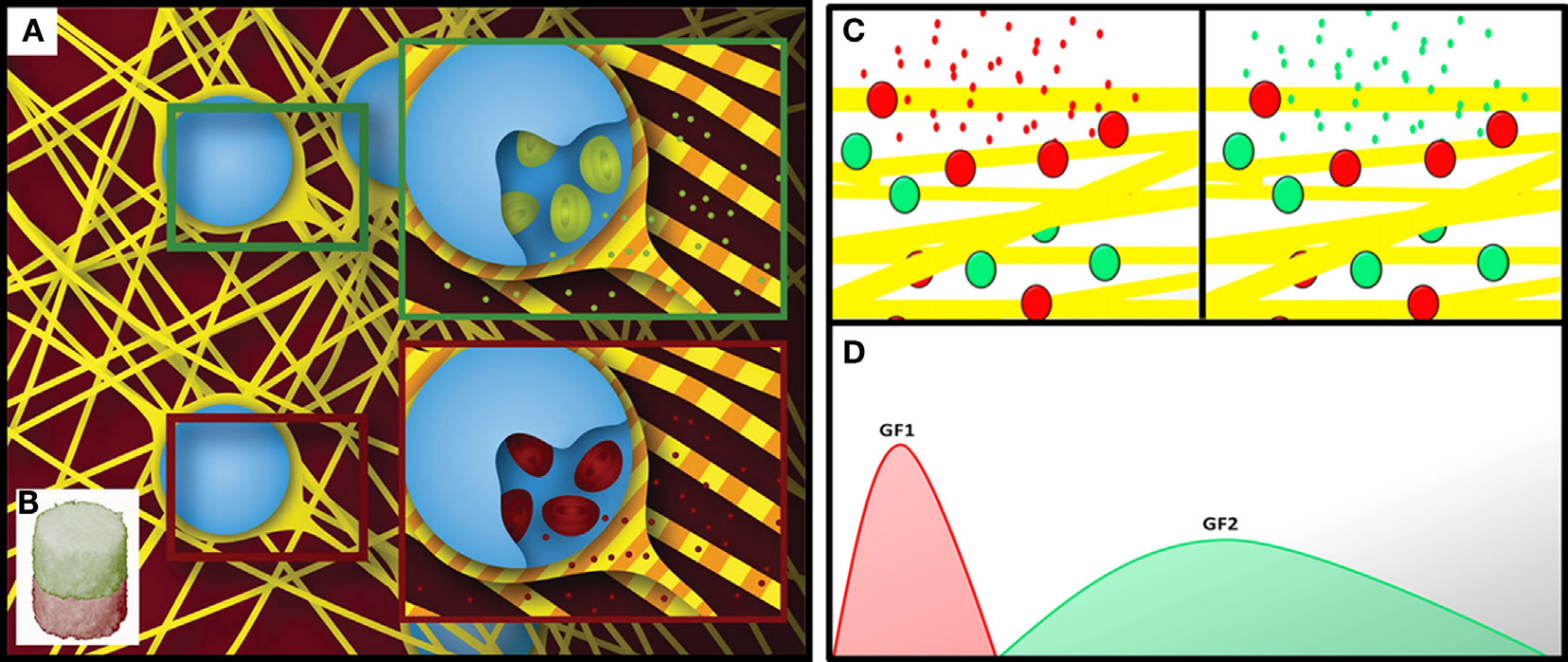
**Schematic showing a spatially patterned fibrous material functionalized with different sets of delivery systems (A), in separate compartments (B) (Minardi et al., 2014)**. Schematic showing the temporal patterning of a material with two sets of delivery systems **(C)**, for the staged release of bioactive molecules **(D)**.

Three-dimensional patterning has been defined as the entrapment of biochemical (Lee et al., 2015a) or structural heterogeneity (Zorlutuna et al., 2011) within the structure of a material and successfully employed in the design of biomimetic materials for tissue engineering. To emulate tissues’ biochemical gradients, the patterning of bioactive molecules within biomaterials should be controlled over time and space (Figure 1).

This has been accomplished using multiple strategies that facilitate controlled spatial and temporal release kinetics.

Hydrogels capable of promoting cell viability and interactions with elements of the ECM have been advantageous for many regenerative medicine applications (Nguyen and West, 2002). Hydrogels are highly hygroscopic polymers that can be fully engineered to mimic specific chemical and physical properties of tissues and are therefore often used as scaffolding materials for tissue engineering applications (Tibbitt and Anseth, 2009). Additionally, due to their biocompatibility and ability to control the release rates of bioactive molecules, hydrogels have been successfully used as drug reservoirs in tissue engineering applications (Peppas et al., 2006). The 3D patterning of hydrogels is crucial to enhance their spatial heterogeneity and improve their features for cell seeding. 3D patterning can fall into two categories: those that integrate patterning during fabrication (i.e., stereolithography) or those that involve post-processing of a uniform hydrogel (Ahmed, 2015). Horn et al. (2007) created hydrogels suitable for spinal cord repair by combining thiol-modified hydroxyl-apatite with acrylate-functionalized poly(ethylene glycol) (PEG). Tsang et al. (2007) developed a multilayer PEG hexagonal hydrogel functionalized with hepatocyte cells and demonstrated better cell viability in the center of the construct compared to uniform hydrogel disks. Lim and Sun (1980) used alginate-based polymers cross-linked with calcium ions for the treatment of diabetic animals. De Souza et al. (2009) demonstrated the *in vivo* biocompatibility of chitosan, phospholipids, and lauric aldehyde or lauric chloride hydrogel blends. Hydrogels can also be patterned by indirect binding. In an elegant recent study, Stupp and coworkers described a successful example of this strategy: they proposed peptide amphiphile nanofibers gels with binding affinity for BMP-2 (Rajangam et al., 2008; Lee et al., 2015b), to solve the health concerns associated with the use of this factor in patients. Others have successfully functionalized the surface of gels and scaffolds with heparin or others glycosaminoglycans to dock bioactive molecules *in situ* (Liang and Kiick, 2014; Corradetti et al., 2016).

One of the major concerns in the engineering of materials functionalized with bioactive molecules for regenerative medicine is the preservation of the payload (Minardi et al., 2016). Due to their structure, 3D scaffolds proved successful in minimizing bioactive factors’ exposure to harsh conditions *in vivo*, preserving their bioactivity (Yuan and Liu, 2012).

Scaffolds have been biochemically patterned through many different approaches. An established strategy consists of the integration of microparticles into the biomaterial structure (Chen et al., 2010). Integrating delivery systems within the scaffold matrix enables the design of scaffolds with patterns of various geometries and purpose, while controlling their release (López-Noriega et al., 2015).

Similarly, our group proposed a chitosan–gelatin scaffold functionalized with composite microspheres consisting of mesoporous silicon microparticles and poly(lactic-co-glycolic acid) (PLGA) for the controlled release of small bioactive molecules (Pandolfi et al., 2016). Wei et al. (2007) showed that incorporating PLGA nanospheres into nanofibrous poly(l-lactic acid) (PLLA) scaffolds loaded with rhBMP-7 enhanced osteogenesis. Kim et al. (2004) accomplished the incorporation of a combination of drugs into a PLGA-based fibrous mat, fabricated through electrospinning.

Interface tissue engineering focuses on the development of tissue grafts capable of replacing defective interfaces, such as ligament-to-bone, tendon-to-bone, and cartilage-to-bone (Almodóvar et al., 2014). These interfaces exhibit anisotropic structural properties, which gradually vary from one tissue to another. Using homogeneous biomaterials ultimately leads to graft failures (Seidi et al., 2011). Singh et al. (2008) introduced an interesting microparticle-based scaffold fabrication technique as a method to create 3D scaffolds with spatial control over multiple bioactive molecules using uniform PLGA microspheres. They demonstrated that embedding the PLGA microparticles into their scaffolds led to more sustained payload release. Recently, we proposed a multiscale approach to selectively integrate different types of nanostructured composite microspheres in a multicompartmented collagen scaffold (Minardi et al., 2014). By fully embedding the microspheres in the type I collagen matrix of the scaffold, the authors were able to spatially pattern the microspheres into the different compartments, and the collagen coating on the microspheres allowed for the zero-order release of the payload for almost 2 months. This method of scaffold functionalization proved to preserve the bioactivity of cytokines for several weeks. The controlled release of the payloads over long periods of time favors on-scaffold cell recruitment while avoiding adverse effects due to the bolus administration of therapeutic molecules in the tissues surrounding the implant (Minardi et al., 2016). To achieve the combined release of multiple molecules from different compartments, Song et al. (2012) incorporated silica nanoparticles into electrospun fibers. Detamore’s group attempted osteochondral regeneration through the creation of continuous gradients of two bioactive factors within a 3D scaffold (Dormer et al., 2010). The authors developed a polymer microsphere-based scaffold, which could be fabricated using microspheres loaded with various GFs to create continuous and opposite gradients of BMP-2 and transforming growth factor-beta (TGF-β) (Mohan et al., 2011).

## Cell Response to 3D Biochemically Patterned Materials

**Figure 2:**
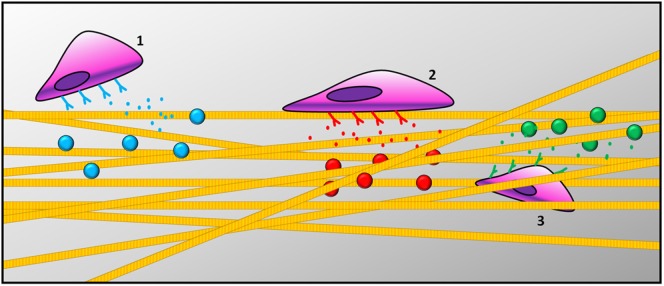
**Schematic representation of cells responding to gradient patterns: (1) cells recruitment, (2) cell adhesion on the surface of the scaffold, and (3) cell migration across scaffold thickness**.

Most of the current understanding of the regenerative process is based on the use of simplified *in vitro* 2D cell cultures, which fail to reproduce tissue complexity. Recently, 3D cultures on biomimetic materials mimicking the ECM microenvironment are becoming the *in vitro* models of choice to study the regenerative process of specific tissues. 3D biomimetic cultures are more physiologically relevant than those in 2D and simpler than *in vivo* models (Hoffmann and West, 2010). As reviewed in the previous paragraph, over the past decade various technologies have been developed to create spatiotemporal gradients for complex biomaterials (Santos et al., 2012). Graded materials can elicit various cell behaviors, such as adhesion, orientation, motility, surface antigen display, cytoskeletal condensation, activation of tyrosine kinases, and modulation of intracellular signaling pathways that regulate transcriptional activity and gene expression (Figure 2). Cells are able to decode information provided by the ECM and respond to specific stimuli, such as topography (Ishii et al., 2005; Raghunathan et al., 2013), mechanical properties (Ulrich et al., 2009; Humphrey et al., 2014), bioactive signals (Li and Folch, 2005; Yañez-Soto et al., 2013; Malik et al., 2015), and concentration gradients of both soluble and tethered GFs (Gattazzo et al., 2014). An established strategy to elicit such biological functions consists of the functionalization of hydrogels with biochemical cues, as described above (Tibbitt and Anseth, 2009; Geckil et al., 2010). Synthetic biomaterials, such as hydrogels or bulk polymeric scaffolds, demonstrated low cellular adhesion, necessitating functionalization with bioactive molecules (Tian et al., 2012). Since synthetic hydrogels lack biochemical cues, different strategies for their 3D biochemical patterning have been developed to increase their level of biomimicry (Luo and Shoichet, 2004) and enhance their interaction with cells (Yan et al., 2011). To favor cell adhesion, migration, and differentiation within hydrogels, the main strategy has been to immobilize adhesive peptides (e.g., RGD) (Bellis, 2011). The use of short peptides to tune cellular response to hydrogels showed success due to the stability of the chemical conjugation between the peptide of interest and the material, without altering its conformation.

Recently, in a very elegant study, the Anseth research group proposed an RGD patterned hydrogel, synthesized through a versatile process of sequential bio-orthogonal click-chemistry reactions (DeForest et al., 2009). Their approach was further developed by García and coworkers, who proposed light to trigger adhesive peptides on the surface of their hydrogel *in vivo* (Lee et al., 2015c). Adhesion and proliferation are not the only two important features to achieve tissue homeostasis, but also cell migration is fundamental for all morphogenetic processes in tissue regeneration (Sternlicht and Werb, 2001). Cell migration occurs in response to gradients of soluble or insoluble signals. In tridimensional constructs, migration is more difficult to achieve due to the mechanical resistance of the surrounding ECM. Synthetic materials must contain cell-adhesive ligands for traction, GFs as signals of migration, as well as space to allow cell movement. Taraballi and colleagues synthesized a plethora of different hydrogels functionalized with bioactive peptides (BMPH1 andBMPH2) that allowed for the adhesion, proliferation, and differentiation of neural stem cells into neurons both *in vitro* and *in vivo* (Gelain et al., 2010, 2011; Taraballi et al., 2010). They demonstrated that neural cells were able to migrate inside the gel but only extended neurites when both factors were presented at the same time. Their findings suggested that several signaling molecules could work best in combination, benefiting from their synergistic effect.

However, in the natural ECM, cells not only respond to signals presented on the surface of the ECM but also to soluble stimuli, especially in the regeneration process. Usually, these factors diffuse through the ECM and bind to their specific receptors on cells’ surface, activating specific transduction cascades. *In vivo*, the transient nature of GF signaling is combined with the slow, sustained signals received from the ECM (Lund et al., 2009). For example, epidermal growth factor (EGF), FGFs, TGF-β, and platelet-derived growth factor (PDGF) (Farokhi et al., 2013) have been used to accelerate wound healing by inducing both epithelial cell and fibroblast proliferation, as well as *de novo* matrix deposition (Kim et al., 2012). Similarly, the release of insulin-like growth factor 1 (IGF-1) and TGF-β1 from polymer scaffolds functionalized with delivery systems showed to successfully induce chondrogenic differentiation (Ertan et al., 2013).

## Conclusion

In the past decade, significant advances have been accomplished in the design of biopatterned materials able to accomplish temporally and/or spatially controlled release of bioactive molecules. Despite these advances, methods should be further developed to prepare patterns and gradients with controlled shape and kinetics, in order to tune the desired cell mechanisms *in vivo*. Altogether, the studies herein reviewed show the potential of 3D biomaterials spatiotemporally patterned with bioactive molecules to recapitulate the complex biochemical *milieu* of target tissues.

## Author Contributions

SM and FT conceived the idea of this review. SM wrote the Abstract, Introduction, and Conclusion sections. LP drafted the first paragraph, FT drafted the second paragraph, and SM and FT revised and finalized the mini-review with the help of ET.

## Conflict of Interest Statement

The authors declare that the research was conducted in the absence of any commercial or financial relationships that could be construed as a potential conflict of interest.
